# Swine Manure Composting With Compound Microbial Inoculants: Removal of Antibiotic Resistance Genes and Their Associations With Microbial Community

**DOI:** 10.3389/fmicb.2020.592592

**Published:** 2020-11-03

**Authors:** Ke Li, Rui Cao, Shangkun Mo, Rensheng Yao, Zhuqing Ren, Jian Wu

**Affiliations:** ^1^College of Animal Sciences and Technology/College of Veterinary Medicine, Huazhong Agricultural University, Wuhan, China; ^2^Animal Husbandry and Veterinary Station of Guangling District, Yangzhou, China

**Keywords:** swine manure, aerobic composting, compound microbial inoculants, antibiotic resistance genes, 16S rRNA sequencing

## Abstract

In this study, compound microbial inoculants, including three *Bacillus* strains and one Yeast strain, were inoculated into swine manure composting to explore the effects on antibiotic resistance genes (ARGs) and mobile genetic elements (MGEs), microbial community structure, and pathogenic bacteria. The results indicated that the abundances of the detected ARGs ranged from 3.6 × 10^3^ to 1.13 × 10^8^ copies/g. The ARGs with the highest abundance was *sul2*, and the lowest was *blaCTX*. Composting removes most of the ARGs and MGEs by 22.8–99.7%. These ARGs were significantly reduced during the thermophilic phase of compost. The removal rate of ARGs at the different layers of compost pile was different as follows: middle layer > upper layer > lower layer. But some ARGs proliferated significantly in the maturation phase of compost, especially the sulfonamide resistance genes. Compound microbial inoculants increased the temperature of compost, accelerated water loss, nitrogen fixation, and increased the removal rate of β-lactamase resistance genes, the transposon gene *tn916* and part of tetracycline resistance genes by 3.7–23.8% in compost. Compound microbial inoculants changed the community structure and increased the *Bacillus* abundance in the thermophilic phase of compost. And it was helpful for removing pathogens during composting. The addition of compound microbial inoculants causes the decrease of *Firmicutes* and the increase of *Bacteroidetes*, which may be related to the removal and proliferation of ARGs.

## Introduction

With the rapid development of intensive culture, antibiotics have been widely used in feed for promoting growth and for disease prevention purposes. However, antibiotics in animal tissues are absorbed incompletely; parts of antibiotics are excreted in urine and feces (Qiu et al., 2016). Antibiotic residues cause serious pollution to the environment, pose a threat to human health, and even have toxic side effects (Sarmah et al., 2006). If the residual antibiotics in the environment do not degrade effectively, these residues may help to maintain or develop antibiotic-resistant microbial populations (Witte, 1998). Additionally, the migration of antibiotics in the environment has led to the spread of resistant genes as a new type of environmental pollutant. Manure carrying antibiotic resistance genes (ARGs) can enter soil, following direct land application (Fang et al., 2015). Many studies have shown that the diversity and abundance of ARGs in soil with long-term application of livestock manure has increased significantly (Ji et al., 2012; Chen et al., 2016), and ARGs in farmland have also increased significantly (Wang et al., 2014; Mu et al., 2015). Therefore, the removal of ARGs from swine manure has emerged as an environmental issue before its application to soil.

Aerobic composting is one of the most effective ways to realize the harmless and resourceful treatment of livestock and poultry manure. Aerobic composting can significantly reduce the concentration of antibiotics in animal manure (Ray et al., 2017), but there is little research on whether ARGs can undergo compost removal. Previous studies show that aerobic composting has various effects on different ARGs (Joy et al., 2014; Su et al., 2015). It has been found that the relative abundances of the tetracycline resistance genes and sulfonamide resistance genes were significantly reduced during the swine manure composting, while quinolone resistance gene still partially remained (Selvam et al., 2012). Thus, composting is most likely an effective way to remove ARGs.

Microbes are the main carriers of ARGs. Several studies have used 16S rRNA sequencing technology to analyze the relationship between ARGs and microbial communities in compost (Jia et al., 2015; Zhang et al., 2016). A large number of studies have shown that exogenous microbial agents can accelerate the composting process and shorten the composting cycle (Nakasaki et al., 2013; Ravindran et al., 2019). However, the current research focuses more on the observation of the application effect of a single functional strain (Zeng et al., 2010). And the impact of inoculation on ARGs is even less studied. Only Duan et al. (2019) studied the effects of *Bacillus subtilis* on ARGs and mobile genetic elements (MGEs) during cattle manure composting. But in practical application, the addition of compound microbial agents is often used to accelerate the composting process. Previous study also showed that compound microbial inoculants can improve composting efficiency of organic solid wastes (Ravindran et al., 2019). Our previous studies have found that *Bacillus* is the main dominant bacterium in the high-temperature period of primitive composting (Yi et al., 2012). Exogenous *Bacillus* spp. may prolong the high-temperature composting time and better kill resistant microorganisms. Thus, in the present study, we delved into the effects of *Bacillus* compound microbial inoculants on composting microbial communities and ARGs. At present, research on these effects has not been reported.

The main aims of the present study were: (1) to investigate the quantitative variation and spatial distribution of ARGs in different periods and different height layers in compost, (2) to use 16S rRNA gene sequencing technology to investigate the dynamic changes of bacterial community structure and composition in compost with the addition of a compound microbial inoculants, and (3) to analyze the correlation between microbial community changes and ARGs. We also detected the relative abundances of pathogenic bacteria during composting. The results of this study may help with the development of high-efficiency microbial agents for the control of ARGs, which is of great significance to guide the application of microbial agents in composting and to further improve composting efficiency.

## Materials and Methods

### Description of Raw Materials

Fresh swine manure was taken from the boutique pig farm of Huazhong Agricultural University. Sawdust from the Veterinary Hospital of Huazhong Agricultural University was approximately 0.5–1 cm in size. The total carbon content of the swine manure was 360.8 g/kg, the total nitrogen content was 30.4 g/kg, and the water content was 69.25%. The total carbon content in sawdust was 471.9 g/kg, the total nitrogen content was 2.2 g/kg, and the water content was 9.38%.

### Composting Process

This composting experiment was conducted in November 2018 at the National Animal Engineering Technology Research Center of Huazhong Agricultural University. Each composting device was a 60-L rectangular foam container (280 × 540 × 400 cm). And it was perforated with one ventilation hole (2 × 2 cm) on the side of the insulation wall. The composting device is very similar to the research from Duan et al. (2019). Two composting experiments were labeled as CK (swine manure + sawdust) and AB (swine manure + sawdust + 1% compound microbial agents − *Bacillus subtilis*: *Bacillus licheniformis*: *Bacillus megaterium*: Yeasts = 1:1:1:2). The initial volume of the compost material is about 50 L, and the fresh weight is about 17 kg. Accordingly, *B. subtilis* have been deposited in China Center for Type Culture Collection (CCTCC) and the CCTCC NO: M2019185. These strains were isolated from previous swine manure composting samples. The C/N of the two experimental materials was adjusted to ⁓20, and the water content was ⁓60%. The composting device was connected to the blower, the blast volume was 5 L/kg∙min, and the ventilation was 5 min per hour.

### Compost Sample Collection

Composting samples were collected individually from the upper, central, and lower portions of the compost to achieve high representativeness of samples in different height layers. Samples collected on days 0 (initial phase), 1 (mesophilic phase), 2 (thermophilic phase), 5 (cooling phase), 12 (maturation phase), and 32 (maturation phase) were designated as detection ARGs samples. At the same time, samples collected on days 1 (mesophilic phase), 2 (thermophilic phase), and 32 (maturation phase) were used to analyze changes in microbial community structure. Each sample was split into two parts: one part was stored at −80°C for subsequent DNA extraction, and another part was stored at 4°C for later chemical analysis.

### Physicochemical Index Analyses

The temperature at the center of each composting container was measured at 9:00 AM daily using a thermometer. At the same time, the room temperature was also recorded daily. Each compost sample and deionized water were mixed at a ratio of 1:10 W(g): V(ml) and shaken at room temperature for 1 h. The pH of the extract was measured. Moisture content was measured by drying in an oven at 105°C for 24 h or until no change in dry weight. Analysis of the total nitrogen (TN), total carbon (TC), and C/N ratios of the samples was performed by using an elemental analyzer (Vario MAX-CN Germany Elementar).

### DNA Extraction and qPCR

Total genomic DNA was extracted using the Fecal DNA Kit (Simgen, Hangzhou, China) according to the manufacturer’s instructions. Nine tetracycline resistance genes (*tetA*, *tetC*, *tetG*, *tetH*, *tetL*, *tetM*, *tetO*, *tetQ*, and *tetW*), five macrolide resistance genes (*ermA*, *ermB*, *ermC*, *ermF*, and *ermQ*), two sulfonamide resistance genes (*sul1* and *sul2*), two β-lactamase resistance genes (*blaCTX* and *blaTEM*), the integrase gene *intI1*, and the transposon gene *tn916* were analyzed by qPCR (Table 1). The qPCR mixtures (total volume, 10 μl) consisted of 5 μl of Hieff qPCR SYBR Green Master Mix (Yeasen, China), 1 μl of template DNA, 0.2 μl of each primer (10 μM), and 3.6 μl of ddH_2_O. qPCR was performed using a LightCycler®96 system (Roche, Switzerland). Each reaction is repeated three times. The qPCR conditions comprised 5 min at 95°C, followed by 40 cycles for 30 s at 95°C, 30 s at the annealing temperatures shown in Table 1, and then 30 s at 72°C. The calibration curve of qPCR was generated using plasmids that carried the target genes. The copy number of ARGs was calculated by the square of the related coefficient (*R*^2^) of the standard curve >0.99 using the external reference method.

**Table 1:** PCR primer sequences and annealing temperature.

Gene	Forward (5'-3')	Reverse (5'-3')	AT(°C)
*16SrRNA*	CCTACGGGAGGCAGCAG	ATTACCGCGGCTGCTGG	55
*tetA*	GCTACTCCTGCTTGCCTTC	CATAGATCGCCGTGAAGAGG	57
*tetC*	GCGGGATATCGTCCATTCCG	GCGTAGAGGATCCACAGGACG	60
*tetG*	GCAGAGCAGGTCGCTGG	CCTGCAAGAGAAGCCAGAAG	57
*tetH*	CAACCCATTACGGTGTGCTA	AAGTGTGGTTGAGAATGCCA	57
*tetL*	TCGTTAGCGTGCTGTCATTC	GTATCCCACCAATGTAGCCG	55
*tetM*	ACAGAAAGCTTATTATATAAC	TGGCGTGTCTATGATGTTCAC	55
*tetO*	GATGGCATACAGGCACAGACC	GCCCAACCTTTTGCTTCACTA	60
*tetQ*	AGAATCTGCTGTTTGCCAGTG	CGGAGTGTCAATGATATTGCA	60
*tetW*	GAGAGCCTGCTATATGCCAGC	GGGCGTATCCACAATGTTAAC	60
*ermA*	AAGCGGTAAACCCCTCTGA	TTCGCAAATCCCTTCTCAAC	57
*ermB*	AAAACTTACCCGCCATACCA	TTTGGCGTGTTTCATTGCTT	57
*ermC*	GAAATCGGCTCAGGAAAAGG	TAGCAAACCCGTATTCCACG	60
*ermF*	TCGTTTTACGGGTCAGCACTT	CAACCAAAGCTGTGTCGTTT	57
*ermQ*	CACCAACTGATATGTGGCTAG	CTAGGCATGGGATGGAAGTC	60
*sul1*	CACCGGAAACATCGCTGCA	AAGTTCCGCCGCAAGGCT	55
*sul2*	GCGCTCAAGGCAGATGGCATT	GCGTTTGATACCGGCACCCGT	60
*blaCTX*	CTATGGCACCACCAACGATA	ACGGCTTTCTGCCTTAGGTT	60
*blaTEM*	TCGGGGAAATGTGCG	GGAATAAGGGCGACA	60
*qnrA*	ATTTCTCACGCCAGGATTTG	GCAGATCGGCATAGCTGAAG	55
*copB*	GGTTGGTCAACAGGATGTCGTACT	TTCCTGCTCGACCAGTTGGAATAC	57
*pcoA*	GCTGCAGATGGCCAGTATGTAAA	CCCTCGAGCGTAACCGGTCC	57
*pcoD*	ATCAGCAGGCAGGACAATAC	CTGATGTGGGTATTAGCTGGATT	55
*czrC*	TAGCCACGATCATAGTCATG	ATCCTTGTTTTCCTTAGTGACTT	60
*czcA*	TCGACGGTGCCGTGGTGATCGTCGAGAA	GTCATCGCCATCGGACGGAACA	60
*tcrB*	CATCACGGTAGCTTTAAGGAGATTTTC	ATAGAGGACTCCGCCACCATTG	55
*intI1*	GGCTTCGTGATGCCTGCTT	CATTCCTGGCCGTGGTTCT	60
*tn916*	TCCTACAGCGACAGCCAGTGA	TGCGTTGCTTTGGTCTGCTGGT	55

### 16S rRNA Gene High-Throughput Sequencing

Six sets of samples collected from two groups, named CK_D1, AB_D1, CK_D2, AB_D2, CK_D32, and AB_D32, were used to identify the microbial communities by performing 16S rRNA gene-based high-throughput sequencing. D1 represents the heating period, D2 represents the high-temperature period, D32 represents the ripening period, and there were three replicates of each group of samples for a total of 18 samples. The V3–V4 hypervariable regions of the bacterial 16S rRNA gene were amplified with primers 338F (5'-ACTCCTACGGGAGGCAGCAG-3') and 806R (5'-GGACTACHVGGGTWTCTAAT-3') by a PCR thermocycler system (GeneAmp 9700, ABI, United States). The high-throughput sequencing method is consistent with Li et al. (2020). Purified amplicons were pooled in equimolar amounts and paired-end sequenced (2 × 300) on an Illumina MiSeq platform (Illumina, San Diego, United States) according to standard protocols by Majorbio Bio-Pharm Technology Co., Ltd. (Shanghai, China).

### Statistical Analysis

GraphPad Prism 7 was used to generate various figures. Averages and SDs of ARGs were determined using Microsoft Excel 2010. The 16S rRNA gene sequencing data were analyzed on the free online I-Sanger Cloud Platform of Majorbio. In addition, we compared the resulting sequences with the constructed Human Pathogenic Bacteria (HPB) database. The HPB database was provided by Shanghai Majorbio Bio-pharm Technology Co., Ltd. The HPB relative abundances were calculated at the genus level.

## Results

### Changes of Conventional Composting Indicators

**Figure 1:**
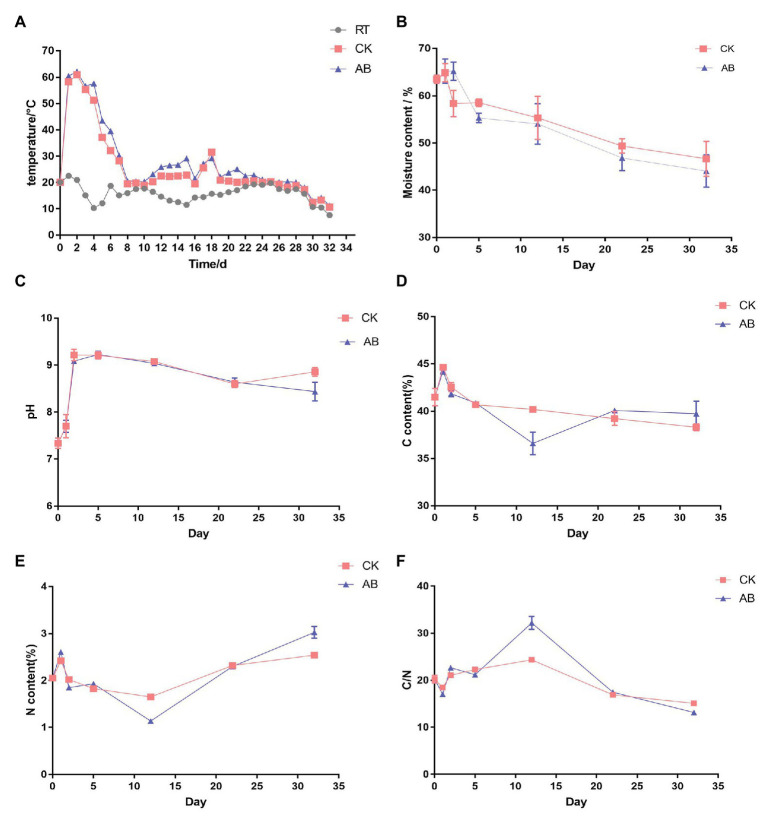
Physicochemical changes during swine manure composting. **(A)** Temperature, **(B)** moisture, **(C)** pH, **(D)** TC, **(E)** TN, and **(F)** C/N in the two treatment conditions during the composting process. CK: the swine manure composting. AB: add 1% compound microbial agents in the swine manure composting.

The key indicators of composting are temperature, pH, moisture, total carbon (TC), total nitrogen (TN), and C/N. The results are shown in Figure 1. Swine manure aerobic composting was performed for 32 days. The maximum temperature in AB was 62.1°C, the maximum temperature in CK was 60.9°C, and the temperatures of the two groups both lasted more than 3 days above 55°C (Figure 1A). It reached the standard of harmless treatment of reactor compost. The moisture content continued to decline during composting and that of the AB group dropped faster than the CK group (Figure 1B). This is because the microbial activity in the AB group compost was stronger, the temperature during the thermophilic phase was higher, and the high temperature lasted longer, so the water loss rate was more rapid. The pH increased gradually in the early stage of composting and declined slowly and stabilized (Figure 1C). In the early stage of composting, the ammonification was strong, the pH value rose rapidly, and the nitrification increased in the maturation phase; then, the pH value began to decrease, and the final pH value was stable between 8 and 9. The TC content in composting process showed a downward trend (Figure 1D). The carbon content of the AB group declined rapidly during the thermophilic phase, which may indicate strong microbial activity in the early stage of composting and converting the organic matter into carbon dioxide or minerals. The change in TN content of the composting piles first decreased and then increased (Figure 1E). The content of TN in the AB group was higher than that in the CK group in the maturation phase, which may be attributable to compound microbial inoculants playing a role in nitrogen preservation. The initial carbon and nitrogen ratio of the material was ⁓20. At the end of composting, the carbon to nitrogen ratio of the AB group was 13.15 and that of the CK group was 15.08 (Figure 1F). Both compost groups reached a mature state. The above results show that adding compound microbial inoculants can increase the maximum temperature of compost, prolong the high-temperature period, and accelerate water loss and nitrogen fixation during swine manure composting. The addition of compound microbial inoculants can accelerate and strengthen the composting process and improve the fertilizer efficiency of composting products.

### Changes of ARGs and MGEs During Composting

**Figure 2:**
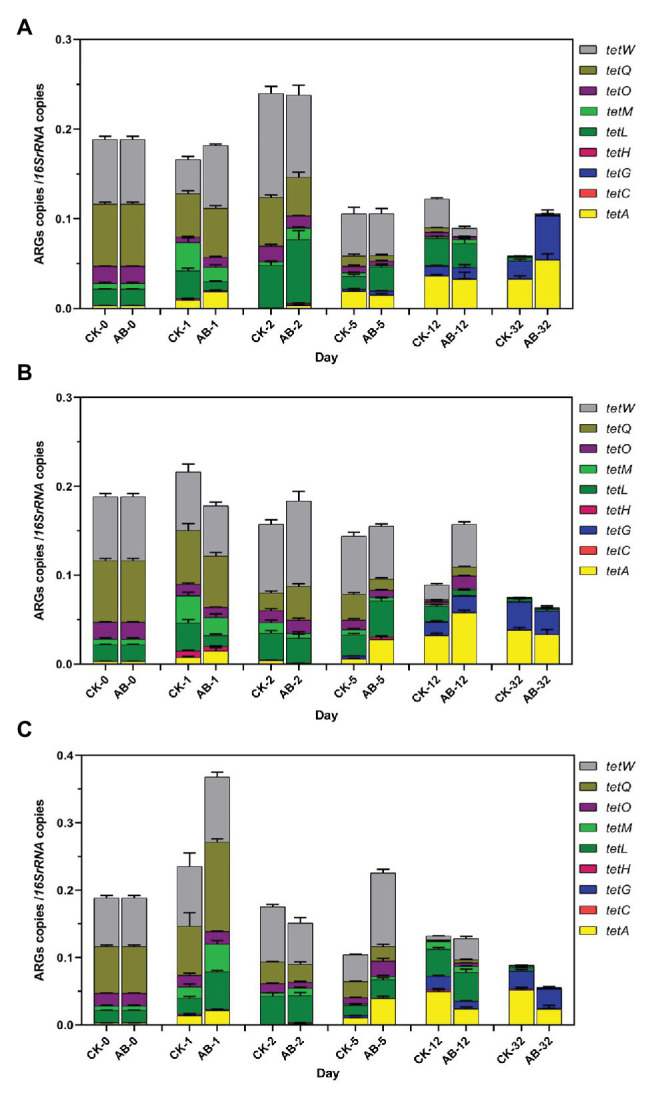
Abundances of tetracycline resistance genes during swine manure composting. **(A–C)** refer to the upper, middle, and lower layers of compost, respectively. CK: the swine manure composting. AB: add 1% compound microbial agents in the swine manure composting. 0, 1, 2, 5, 12, and 32 are the days for composting.

**Figure 3:**
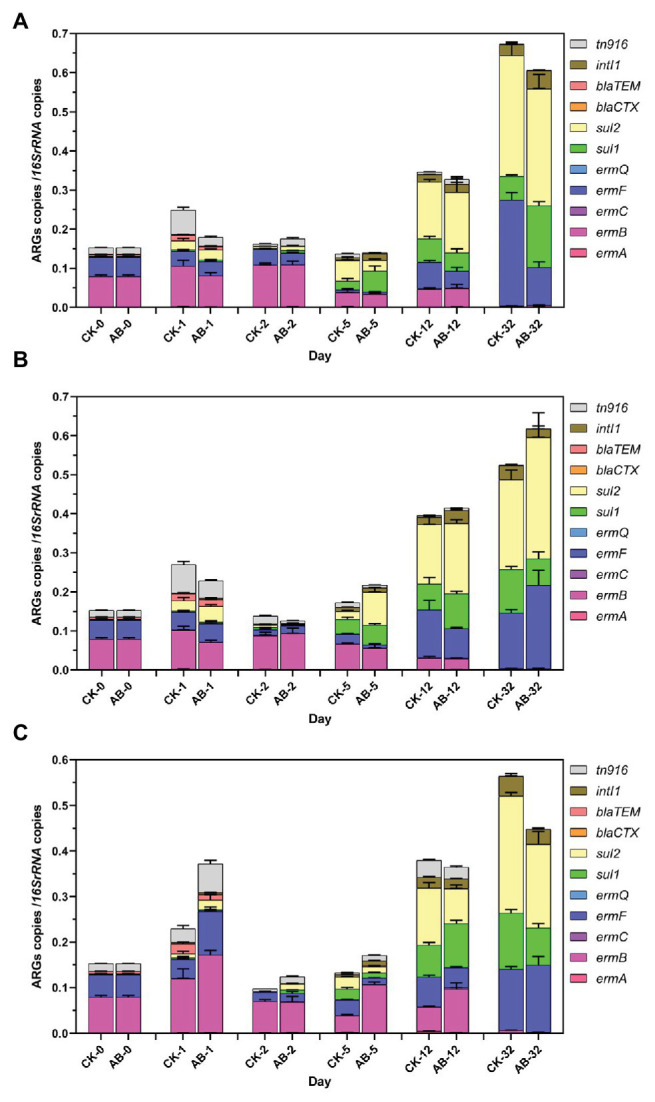
Abundances of ARGs and MGEs during swine manure composting. **(A–C)** refer to the upper, middle, and lower layers of compost, respectively. CK: the swine manure composting. AB: add 1% compound microbial agents in the swine manure composting. 0, 1, 2, 5, 12, and 32 are the days for composting.

Eighteen ARGs and two MGEs were detected during swine manure composting. ARGs showed the same trend in the two groups (Figures 2, 3). The abundances of ARGs ranged from 3.6 × 10^3^ to 1.13 × 10^8^ copies/g. The ARGs with the highest abundance was *sul2*, and the lowest was *blaCTX*. For nine tetracycline resistance genes, the abundance of *tetW* gene is the highest, and the *tetG* abundance is the lowest (Figure 2). As the compost goes on, the reservoir of 11 ARGs (*tetH*, t*etL*, t*etM*, *tetO*, *tetQ*, *tetW*, *ermB*, *ermQ*, *blaCTX*, *blaTEM*, and *tn916*) are reduced at the end of composting. The total removal rates are 22.8–99.7%. *tetM*, tetO, *tetQ*, *ermB*, *ermQ*, and *blaTEM* were significantly reduced at the end of the thermophilic phase on the 5th day. At the end of composting, the reduction effect of ARGs (*tetH*, *tetL*, *tetM*, *blaCTX*, and *tn916*) in the AB group was significantly higher than that in the CK group. Compound microbial inoculants increased these ARGs removal in swine manure with increasing rates of 3.7–23.8%. At the same time, we also studied the spatial distribution and variation characteristics of ARGs during composting. We found that the contents of ARGs at different layers of the composting piles were significantly different. The abundance of ARGs in the middle and lower layers of compost showed the same trend. Overall, the reduction effect of ARGs was as follows: middle layer > upper layer > lower layer. On the other hand, we found that *tetA*, *tetC*, *tetG*, *ermA*, *ermC*, *ermF*, *sul1*, *sul2*, and *intI1* proliferated. These ARGs began to proliferate on the 5th day, and the abundance increased significantly on the 12th day. This may be due to the proliferation of bacteria carrying these ARGs during the maturation phase.

### Variation of Microbial Community

**Figure 4:**
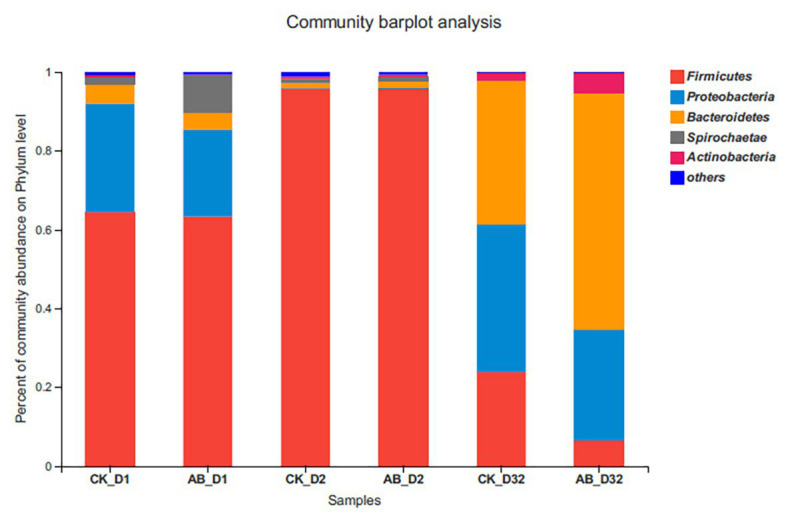
Structure and abundance of flora at the phylum level. CK: the swine manure composting. AB: add 1% compound microbial agents in the swine manure composting. D1 represents the mesophilic phase, D2 represents the thermophilic phase, and D32 represents the maturation phase.

Change of microbial community at the phylum level during composting is shown in Figure 4. The sequences data reported in this study have been deposited in NCBI SRA with the accession number PRJNA663659 (https://www.ncbi.nlm.nih.gov/sra/; PRJNA663659). *Firmicutes*, *Actinobacteria*, *Bacteroidetes*, *Spirochaetae*, and *Proteobacteria* were the most dominant flora during the two kinds of composting. However, their relative abundances varied significantly at different phases of composting (*p* < 0.05). There was no significant difference in the structural composition of AB and CK between the mesophilic phase and thermophilic phase. *Firmicutes* and *Proteobacteria* were dominant flora during the mesophilic phase. However, *Firmicutes* accounted for more than 95% during the thermophilic phase. In the maturation phase, there was a significant difference in community structure between AB and CK. *Bacteroidetes* and *Actinobacteria* in group AB were 23.8 and 3.1% higher than those in group CK, respectively. But *Firmicutes* and *Proteobacteria* in group AB were 17.3 and 9.5% lower than those in group CK, respectively.

**Figure 5:**
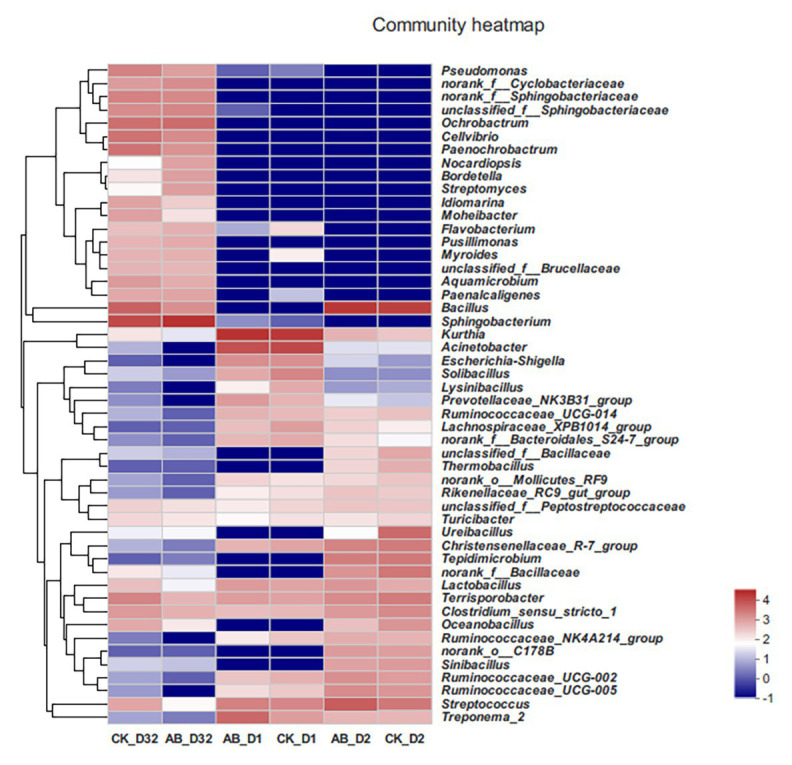
Heat map of species abundance at the genus level in compost. CK: the swine manure composting. AB: add 1% compound microbial agents in the swine manure composting. D1 represents the mesophilic phase, D2 represents the thermophilic phase, and D32 represents the maturation phase.

At the same time, we also analyzed the changes of microbial communities during composting at the genus level (Figure 5). In the mesophilic phase, the composition of CK and AB flora was similar. *Kurthia* showed the highest abundance, followed by *Acinetobacter*, *Escherichia*, *Shigella*, *Streptococcus*, and *Treponema*. In the thermophilic phase (day 2), *Bacillus* showed the highest abundance. The *Bacillus* abundance of the AB group was higher than that of the CK group, which may be due to the addition of compound microbial inoculants. At the end of composting, the abundance of *Bacillus* dropped dramatically, replaced by *Sphingobacterium*, *Ochrobactrum*, *Cellvibrio*, etc. From the results, the microbial community structure at different stages of the composting was quite different. The addition of compound microbial inoculants changed the community structure during composting.

### Variation of Pathogens During Composting

**Figure 6:**
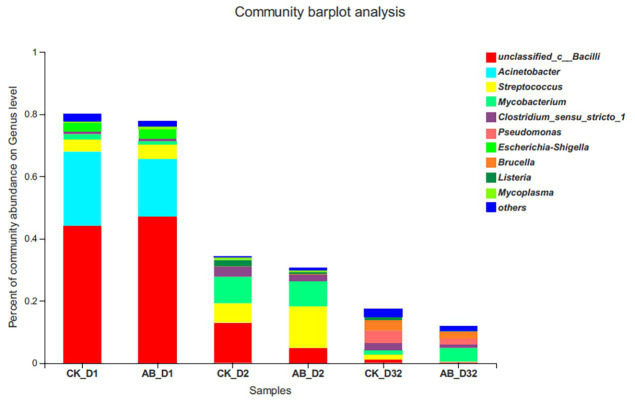
Relative abundance changes of human pathogenic bacteria (HPB) during composting. CK: the swine manure composting. AB: add 1% compound microbial agents in the swine manure composting. D1 represents the mesophilic phase, D2 represents the thermophilic phase, and D32 represents the maturation phase.

We analyzed the change of HPB relative abundance during the composting process (Figure 6). HPB assigned to 10 genera were detected during composting, among which *Unclassified Bacilli*, *Streptococcus*, *Acinetobacter*, and *Escherichia/Shigella* were dominant. The abundance of pathogens decreased sharply during the thermophilic phase on the 2nd day of composting. At the end of composting, the relative abundance of HPB in the CK group decreased by 78.16%, and the AB group decreased by 86.77%. At the end of composting, the composition of composting bacteria in the two groups was different. The CK group mainly consisted of *Pseudomonas* and *Brucella*, while the AB group was mainly *Mycobacterium*.

### Associations of Bacterial Community With Environmental Factors

**Figure 7:**
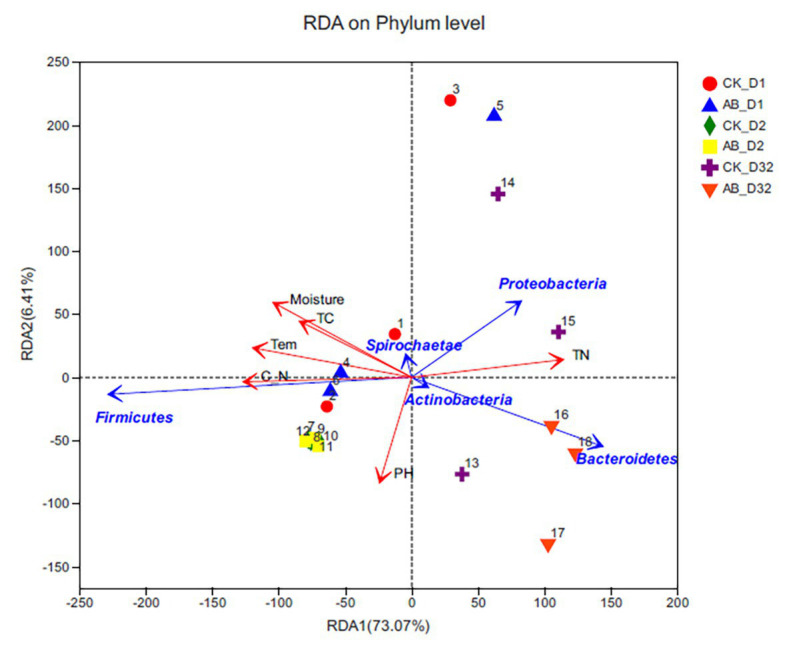
Redundancy analysis between environmental factors and microbial community structure (phylum level). CK: the swine manure composting. AB: add 1% compound microbial agents in the swine manure composting. D1 represents the mesophilic phase, D2 represents the thermophilic phase, and D32 represents the maturation phase.

CCA analysis is mainly used to reflect the correlation between bacterial community and environmental factors (Figure 7). The results showed that the selected environmental variables explained a total of 79.48% of species changes. Temperature, moisture, TC, TN, and C/N were significantly correlated with microbial communities, and pH had little effect on the community. *Actinobacteria* and *Tenericutes* were more affected by environmental factors. The change of TN content had a greater influence on *Proteobacteria* and *Bacteroidetes*. Temperature and C/N changes were closely related to *Firmicutes*. Combined with the stage of composting, TC, and moisture played important roles in mesophilic phase. At the thermophilic phase, temperature and C/N played important roles. During the maturation phase, TN and pH affected microbial community changes.

### Correlations Between ARGs, MGEs, and Microbial Community

**Figure 8:**
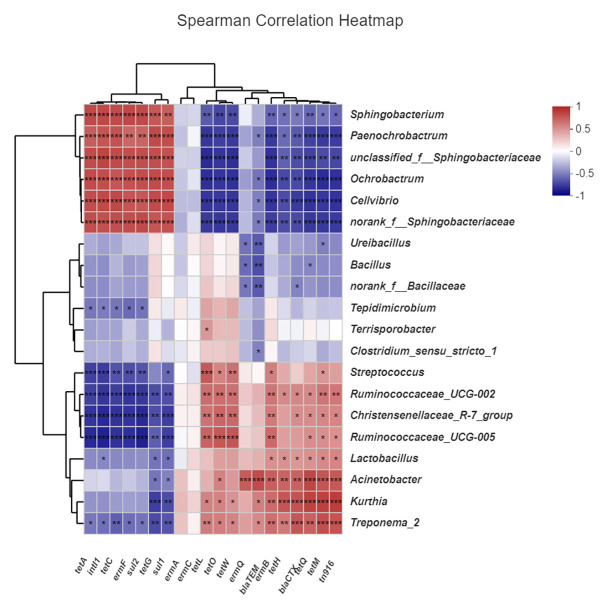
Correlation between antibiotic resistance genes (ARGs), mobile genetic elements (MGEs), and microbial community. CK: the swine manure composting. AB: add 1% compound microbial agents in the swine manure composting. D1 represents the mesophilic phase, D2 represents the thermophilic phase, and D32 represents the maturation phase.

We analyzed the correlations between ARGs, MGEs, and microbial community structure (genus level, top 20 abundances). As shown in Figure 8, *tetL*, *tetO*, *tetW*, and *intI1* were significantly positively correlated with *Streptococcus*, *Christensenellaceae*, and *Ruminococcaceae*. *ermQ* and *blaTEM* were significantly positively correlated with *Acinetobacter*. *ermB*, *tetH*, *blaCTX*, *tetQ*, *tetM*, and *tn916* were significantly positively correlated with *Acinetobacter*, *Kurthia*, and *Treponema_2*. These microflora occupy a dominant position in the early stage of composting and were greatly reduced in the maturation phase, so these ARGs were also reduced. On the other hand, *tetA*, *tetC*, *tetG*, *ermF*, *sul1*, and *sul2* were significantly positively correlated with *Sphingobacterium*, *Paenochrobactrum*, *Ochrobactrum*, and *Cellvibrio*. These microflora occupy a dominant position during the maturation phase of composting, so these ARGs proliferate at the end of composting. However, *ermA* and *ermC* failed to match the related flora, and possibly because of the low abundance of host bacteria, they could not enter the forefront of species matching.

## Discussion

Composting is an effective way to remove ARGs. Some ARGs were significantly reduced during the thermophilic phase. This demonstrated that thermophilic phase of aerobic composting plays important roles in the removal of ARGs, which is consistent with the previous studies (Cui et al., 2016; Zhang et al., 2016, 2020). But some ARGs proliferated significantly in the late period of composting. Our results show that three tetracycline ARGs, three macrolide ARGs, and two sulfonamide ARGs and *intI1* proliferated during the late composting period. It has been found that *intI1* is closely related to macrolide ARGs and sulfonamide ARGs (Cheng et al., 2013; Su et al., 2015). So the proliferation of ARGs may be involved in the *intI1*-mediated HGT (Horizontal gene transfer) process.

This study found that the addition of compound microbial inoculants could remove more ARGs and MGEs than natural composting. It could be that the compound microbial inoculants may kill the host bacteria carrying ARGs or high temperature inhibited HGT and eliminated some ARG hosts. However, composting process had a greater impact on ARGs than compound microbial inoculants. In this study, the composting process removes most of ARGs and MGEs by 22.8–99.7%, but compound microbial inoculants only increased the removal rate of part of ARGs by 3.7–23.8% in compost. This is consistent with the research results of Duan et al. (2019). And composting could not remove the ARGs completely. Some ARGs showed more proliferation in the AB group than in the CK group at the end of composting. This may be related to the change of the community structure of compost with the addition of compound microbial inoculants. Microorganisms carrying ARGs proliferate in large numbers in the maturation phase of composting. Some *thermophilic* bacteria may be the hosts of ARGs (Qian et al., 2016).

Similar to Awasthi et al. (2019) and Wu et al. (2020), microbial community composition was the most direct factor affecting ARGs during composting. The richness and diversity of microbial communities in the composting process showed a downward trend in the maturation phase of composting, which also explained why most ARGs were reduced. First, the decrease of microbial density constrains the horizontal transfer process of ARGs (Ma et al., 2016). Second, due to the decrease of bacterial diversity, the host bacteria of ARGs were also reduced (Wang et al., 2017). At the same time, our research also indicated that the relative abundance of *Firmicutes* decreased, but *Bacteroidetes*, *Proteobacteria* and *Actinobacteria* were increased in the maturation phase of compost. Duan et al. (2019) pointed out that *Firmicutes* are the main cause of ARGs changes during cattle manure composting. It has been reported that a decrease in the relative abundance of *Firmicutes* might explain the removal of most ARGs in compost (Huerta et al., 2013; Stegmann et al., 2015). Luo et al. (2017) showed that more than 50% of potential hosts of ARGs and MGEs belonged to *Bacteroidetes* and *Proteobacteria*. Zhang et al. (2020) indicated that *Actinobacteria* were the primary potential host of ARGs during commercial livestock manure composting. It follows that the proliferation of some ARGs may be related to the increase of *Bacteroidetes*, *Proteobacteria*, and *Actinobacteria* in maturation phase. The analysis at the genus level verifies the above conclusions. The abundance of *Bacillus* has decreased significantly, and it has been replaced by *Sphingobacterium*, *Ochrobactrum*, *Cellvibrio*, etc., in maturation phase, which are closely related to the proliferation of part ARGs. These bacteria belong to *Bacteroidetes* or *Proteobacteria*.

The addition of compound microbial inoculants altered the microbial community in the compost. The *Bacillus* abundance was higher in the thermophilic phase than that of the control group. *Bacillus* is the most important component in the compound microbial inoculants. This also directly proves the effect and experimental reliability of adding compound microbial inoculants. At the same time, our research also indicated that the relative abundance of *Firmicutes* and *Proteobacteria* decreased, but *Bacteroidetes* and *Actinobacteria* were increased in the maturation phase of compost by adding compound microbial inoculants. Among them, *Firmicutes* and *Bacteroidetes* change greatly. The succession of *Firmicutes* and *Bacteroidetes* during the composting may be the potential driver for ARGs alteration. The relative abundance of *Firmicutes* in group AB was significantly lower than that in group CK. This also explains that the removal of ARGs in the AB group was higher than that in the CK group.

Most environmental factors were significantly correlated with microbial community. Zhang et al. (2019) pointed out that environmental factors may not directly affect ARGs, but may indirectly affect the changes of ARGs by influencing microbial community. Duan et al. (2019) found *B. subtilis* at 0.5% could cause a change in compost’s pH, thereby reducing ARGs during cattle manure composting. However, there was no significant difference in pH between the two groups in this study, which had little impact on the microbial community. We also found that there were large numbers of pathogens in the compost material by analyzed the changes of microbial communities at the genus level. Previous study has also shown that most of the host bacteria of ARGs are pathogens (Li et al., 2015). Our results showed that compost removes most of the pathogens and the addition of compound microbial inoculants helped with the removal of pathogens. Fan et al. (2020) also found the relative abundance of potential HPB decreased by 74.6 and 91.4% at the end of vacuum-type composting and positive-pressure composting, respectively. But a recent study shows that the total relative abundance of pathogens did not obviously decline with the composting progressing during commercial livestock manure composting (Zhang et al., 2020). This result is inconsistent with ours.

## Conclusion

Composting removes most of the ARGs and MGEs. ARGs were significantly reduced during the thermophilic phase, and the reduction effect of ARGs at the different layer of compost pile was as follows: middle layer > upper layer > lower layer. But some ARGs proliferated significantly in the maturation phase of composting, especially the sulfonamide resistance genes. Compound microbial inoculants increased the maximum temperature of compost, accelerated water loss and nitrogen fixation, and changed the community structure during swine manure composting. Inocula increased the removal rate of β-lactamase resistance genes, the transposon gene *tn916*, and part of tetracycline resistance genes in compost. The dominant phyla were *Firmicutes*, *Proteobacteria*, and *Bacteroidetes* in swine manure compost. ARGs were significantly positively correlated with microbial communities. Compound microbial inoculants changed the community structure and were helpful for removing pathogens during composting.

## Data Availability Statement

The sequences data reported in this study have been deposited in NCBI SRA with the accession number PRJNA663659 (https://www.ncbi.nlm.nih.gov/sra/PRJNA663659).

## Author Contributions

JW and KL contributed to the study conception and design. KL, RC, SM, and RY conducted the experiments and analyzed data. The first draft of the manuscript was written by KL and JW. All authors contributed to the article and approved the submitted version.

### Conflict of Interest

The authors declare that the research was conducted in the absence of any commercial or financial relationships that could be construed as a potential conflict of interest.
